# Romosozumab added to ongoing denosumab in postmenopausal osteoporosis, a prospective observational study

**DOI:** 10.1093/jbmrpl/ziae016

**Published:** 2024-02-07

**Authors:** Giovanni Adami, Elisa Pedrollo, Maurizio Rossini, Angelo Fassio, Vania Braga, Emma Pasetto, Francesco Pollastri, Camilla Benini, Ombretta Viapiana, Davide Gatti

**Affiliations:** Rheumatology Unit, Department of Medicine, University of Verona, Verona, 37134, Italy; Rheumatology Unit, Department of Medicine, University of Verona, Verona, 37134, Italy; Rheumatology Unit, Department of Medicine, University of Verona, Verona, 37134, Italy; Rheumatology Unit, Department of Medicine, University of Verona, Verona, 37134, Italy; Rheumatology Unit, Department of Medicine, University of Verona, Verona, 37134, Italy; Rheumatology Unit, Department of Medicine, University of Verona, Verona, 37134, Italy; Rheumatology Unit, Department of Medicine, University of Verona, Verona, 37134, Italy; Rheumatology Unit, Department of Medicine, University of Verona, Verona, 37134, Italy; Rheumatology Unit, Department of Medicine, University of Verona, Verona, 37134, Italy; Rheumatology Unit, Department of Medicine, University of Verona, Verona, 37134, Italy

**Keywords:** romosozumab, denosumab, bone mineral density (BMD), bone turnover markers (BTMs), bone metabolism

## Abstract

**Background:**

Optimization of sequential and combination treatment is crucial in shaping long-term management of postmenopausal osteoporosis (OP).

**Methods:**

We conducted a 6-month prospective observational study on postmenopausal women with severe OP receiving treatment with romosozumab either alone (in patients naïve to treatment) or in combination with ongoing long-term denosumab (>2 years) or continuing ongoing denosumab alone (>2 years). We collected serum samples for bone turnover markers, bone modulators, and calcium phosphate metabolism at baseline, month 3 and month 6. BMD was assessed at baseline and after 6 months.

**Results:**

Fifty-two postmenopausal women with OP were included in the study. Nineteen received romosozumab alone, 11 received romosozumab combined to ongoing denosumab, and 22 continued denosumab alone. BMD increased significantly at all sites at 6 months of follow-up in the romosozumab alone group (femoral neck +8.1%, total hip +6.8%, and lumbar spine +7.9%). In contrast, BMD increased significantly only at lumbar spine in the combination group (+7.2%) and in the denosumab group (+1.5%). P1nP increased significantly in romosozumab groups at month 3 (+70.4% in romosozumab alone group and +99.1% in combination group). Sclerostin levels increased steeply in both romosozumab groups, and Dkk1 did not change.

**Conclusion:**

Romosozumab added to ongoing denosumab resulted in an increase in P1nP and lumbar spine BMD, but not in femoral neck BMD. For patients on denosumab, using romosozumab as an additional treatment appeared to be useful in terms of bone formation markers and spine BMD vs denosumab alone. Further randomized controlled trials, possibly powered to fracture outcomes, are needed to confirm our results.

## Graphical Abstract

**Graphical Abstract:**
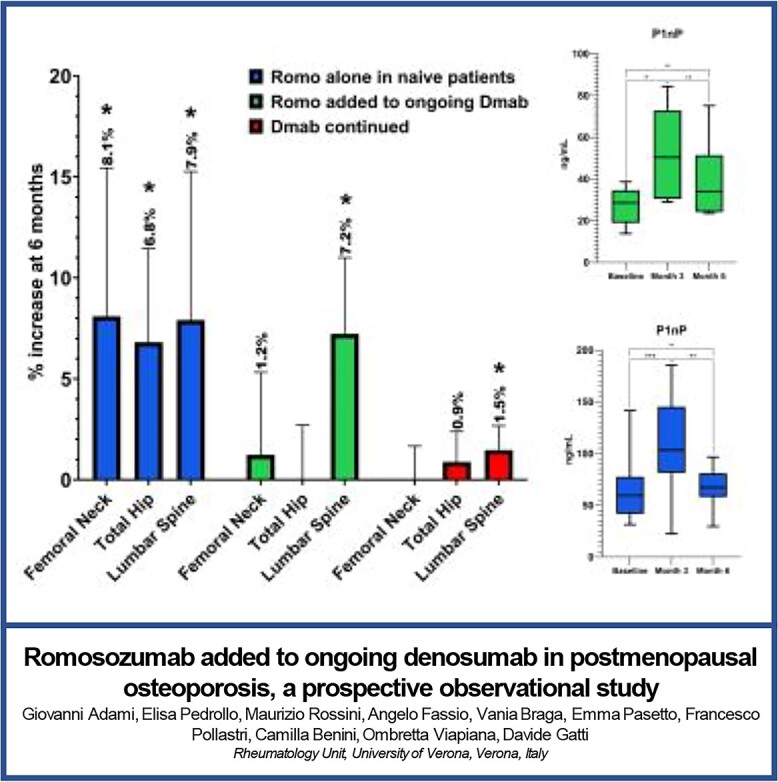

## Introduction

Current therapies approved for postmenopausal osteoporosis (OP) include antiresorptive drugs (bisphosphonates and denosumab), anabolic drugs (teriparatide and abaloparatide), and romosozumab (an anti-sclerostin monoclonal antibody with a dual action on both bone formation and bone resorption).^1^ Histomorphometry studies showed that teriparatide and abaloparatide increase bone density leveraging mainly on remodeling bone formation and overflow remodeling bone formation.^2–4^ In contrast, romosozumab has been shown to increase mainly modeling-based bone formation and only partially remodeling-based bone formation.^5^

Treatment regimens can include sequential use of drugs with shared or different modes of action.^6^ One of the main reasons to change a treatment regimen is treatment failure, which should be considered if a fragility fracture, a notable loss of BMD, or non-suppression of bone metabolism is observed. However, while switching from a bisphosphonate to a bone anabolic has been proven to be safe and effective (albeit not perfectly optimal),^6–8^ the transition from denosumab to teriparatide may expose patients to an increased fracture risk due to the synchronous excess activation of multiple bone remodeling units at the time of loss-of-effect (the so-called rebound effect).^9^^,^^10^ This phenomenon has been described in the DATA-switch study,^11^ in which the transition from denosumab to teriparatide resulted in rapid bone loss, especially at cortical sites (femur).

Romosozumab, when administered after long-term alendronate (STRUCTURE study),^12^ showed lower BMD gains compared to when given in naive patients (ARCH and FRAME studies).^13^^,^^14^ Interestingly, romosozumab given after 1 year of denosumab (STRUCTURE study Phase 2 extension) resulted with minimal to no BMD gain at the hip.^15^

In summary, sequential treatment with denosumab followed by teriparatide or romosozumab appeared to be detrimental or at least sub-optimal in the case of romosozumab.^16^

The combination of teriparatide and denosumab in naïve patients with postmenopausal OP was studied the DATA study.^11^ This study showed greater increase in BMD at the femoral neck, total hip, and spine with the combination therapy compared with either drug alone.

To our knowledge, no study has explored the effect of combination of romosozumab and denosumab. The objective of the present study is to describe the effects of adding romosozumab to ongoing denosumab treatment, in comparison with romosozumab or denosumab alone.

## Material and methods

We conducted a 6-month observational prospective study on women with OP starting treatment with monthly subcutaneous injections of romosozumab (at a dose of 210 mg/month) naïve to treatment or added to patients with ongoing denosumab treatment (>2 years of continuing treatment):

*Inclusion criteria:*

- Post-menopausal women with documented severe osteoporosis 10-year major osteoporotic fracture (MOF) fracture risk ≥20% (determined with DeFRA tool, a validated fracture risk assessment tool derived from FRAX^17–20^) plus
- T-score at the spine or femur of less than −2.5 (or less than −2.0 if there were ≥2 moderate or severe vertebral fractures or if there was a femoral fracture in the previous 2 years) and history (ie, at any time) of ≥1 moderate or severe vertebral fractures or ≥2 mild vertebral fractures or ≥1 femoral fracture or
- History (ie, at any time) of ≥2 non-vertebral non-femoral fractures (including non-vertebral, non-femoral MOF, and non-MOF fractures)

*Exclusion criteria:*

- History of previous myocardial infarction or stroke
- History of bone diseases other than osteoporosis (eg, Paget’s disease of the bone)
- History of malignancy of the bone
- Severe liver or kidney disease (estimated glomerular filtration rate (eGFR) <30 mL/min or Child–Pugh grade B or C)
- Uncontrolled endocrine disease (ie, hypocalcemia, primary hyperparathyroidism
- Treatment with bisphosphonates for >6 months in the previous 12 months of romosozumab initiation

In patients on denosumab experiencing a fracture while on treatment, the decision to add romosozumab to denosumab was deemed by the clinician and not based on formal inclusion or exclusion criteria apart from the criteria listed above. However, we tended to focus on MOF and we did not prescribe romosozumab in patients with non-MOF fractures.

We also included a group of patients with ongoing denosumab as a control group to isolate the specific effects attributable to romosozumab, especially on bone turnover markers (BTMs) and calcium metabolism. Moreover, since denosumab has a non-plateaued effect on BMD; by including the denosumab-only group, we might have the opportunity to compare the magnitude of the effect of denosumab alone with the combination of the two drugs.

BMD measurements were taken, at baseline and after 6 months of therapy, at the femoral neck, total hip, and lumbar spine (L1–L4) using dual-energy X-ray absorptiometry (DXA) with the QDR Hologic Delphi machine. The variation coefficient for the vertebral site was 1%, while it was 1.2% for the femoral neck. Vertebral fracture assessment (VFA) was performed to assess for new radiological vertebral fractures. Blood samples were collected in the morning after fasting at baseline, month 3 and month 6. The serum samples were aliquoted and stored at −80°C until they were assayed for various markers. These markers included the C-terminal telopeptide of type I collagen (CTX, a marker of bone resorption), Procollagen I Intact N-Terminal Peptide (P1NP, a marker of bone formation), Dkk1 (a Wnt inhibitor), sclerostin (a Wnt inhibitor), 25OH-Vitamin D (25OHVitD), and parathyroid hormone (PTH). The measurements of CTX and P1NP were performed using the IDS-ISYS Multi-Discipline Automated Analyzer based on chemiluminescence technology. The intra-assay coefficients of variation were 3.0% for P1NP and 2.0% for CTX. Serum Dkk1 and sclerostin were measured using ELISA kits, with sensitivities of 0.89 and 8.9 pmol/L, respectively, and intra-assay coefficients of variation of 7.8% and 5.6%, respectively. The inter-assay variabilities were 8.2% and 6.9% for Dkk1 and sclerostin, respectively. PTH was measured using ELISA with an intra-assay variability of 6% and an inter-assay variability of 7%. 25OHVitD was measured using the LIAISON 25OHVitD assay, with an intra-assay variability of 8% and an inter-assay variability of 12%. To minimize inter-assay variability, all samples were measured in a single batch.

Group comparisons were performed with analysis of variance (ANOVA) post-hoc tests. Categorical variables were compared with the *χ*^2^ test. All differences were considered significant when the *P*-value was inferior to 0.05.

We analyzed BMD and serum markers changes with mixed-effect model analysis for repeated measures. If there are missing values in the data, repeated measures ANOVA cannot be used. Instead, we utilized GraphPad Prism 9.5.1 to analyze the data by fitting a mixed model that employs a compound symmetry covariance matrix, with a restricted maximum likelihood approach. It's worth noting that in the absence of missing values, this approach provides the same *P*-values and multiple comparisons tests as the repeated measures ANOVA. We adjusted *P*-values in multiple comparisons using Tukey's procedure. The changes in BMD between groups, from baseline, were examined using a repeated measures model. This model adjusted for several factors as fixed factors: treatment, age, and the initial BMD. Associations between continuous variables were tested using Pearson correlation coefficients. To account for multiplicity, we used the false discovery rate (FDR) approach with the two-stage step-up method of Benjamini, Krieger, and Yekutieli (*Q* value 5% of FDR). All statistical analyses were performed using SPSS Version 26 (SPSS, Inc., Chicago, IL, USA) and GraphPad Prism version 9.5.1 (GraphPad Software, San Diego, CA, USA). This study was approved by the University of Verona ethic committee (prot. registration: REUMABANK). All patients provided informed consent to participate in the study.

## Results

Fifty-two postmenopausal women were included in the study. The mean age was 72.9 ± 9.4 years, and the mean age at menopause was 49.4 ± 3.1 years. The majority the study population had a history of at least one vertebral fracture (92%, *n* = 48), with median of 2 (IQR 1.25–3) vertebral fractures per patient; 61% of the patients (*n* = 32) had a history of non-vertebral MOF fractures, and 44% (*n* = 23) of patients had a history of non-MOF.

Twenty-six patients had at least one cardiovascular risk factor (arterial hypertension or dyslipidemia). Twelve (23%) patients had not severe chronic kidney disease, with eGFR >45 mL/min, and 2 patients had a monoclonal gammopathy. The baseline T scores were −2.6 ± 0.8, −2.6 ± 0.9, and −2.7 ± 1 at femoral neck, total hip, and lumbar spine, respectively.

We then stratified the overall population into the

- “Romosozumab alone” group (*n* = 19, 63%): patients naïve to treatment who did not tolerate oral bisphosphonates for adverse events. In detail: all patients received <3 months of oral bisphosphonates, >6 months prior to romosozumab initiation. Ten patients received oral alendronate and reported gastrointestinal complaint (nausea, pain, or constipation), 5 patients received oral risedronate and reported gastrointestinal complaint (nausea, pain, and a case of gastric bleeding), and 4 patients received oral ibandronate and reported gastrointestinal complaint (nausea, pain, or constipation).
- “Combination” group (*n* = 11, 37%): patients treated with long-term denosumab (>2 years of ongoing denosumab, median treatment 4 years, IQR 3–5) in whom romosozumab was started in combination with ongoing denosumab (ie, denosumab 60 mg injection within 3 weeks from monthly 210 mg romosozumab initiation). All these patients had a vertebral fracture while on denosumab. Seven had a new radiographic vertebral fracture, and four had a new worsening of a prevalent vertebral fracture.
- “Denosumab alone” group in whom ongoing denosumab (>2 years of ongoing denosumab, median treatment 3 years, IQR 2–4) was continued.

We did not find any significant difference in baseline characteristics between groups except for slightly higher BMD levels at lumbar spine in the denosumab alone group compared to the other two groups and higher BMD at total hip in the denosumab alone group vs romosozumab alone group. Table 1 shows the baseline characteristics of the study population stratified by treatment.

**Table 1:** Baseline study population characteristics.

Cohort characteristics	Romosozumab alone (*n* = 19)	Romosozumab + denosumab (*n* = 11)	Denosumab alone (*n* = 22)	*P*-value
Age ± SD—yr	73.5 ± 9.6	71.7 ± 9.9	73.2 ± 9.1	ns
Age at menopause ± SD—yr	49.4 ± 4.2	50.3 ± 4.2	48.9 ± 4.1	ns
Prevalent vertebral fx—*n* (%)	17 (89)	11 (100)	20 (90)	ns
Recent vertebral fx (<2 yrs)—*n* (%)	7 (37)	11 (100)	3 (14)	^a^
Median *n* vertebral fractures (IQR)	2 (1–3)	3 (2–4)	2 (1–3)	ns
Prevalent non-vertebral MOF fractures—*n* (%)	12 (63)	7 (64)	13 (59)	ns
Recent non-vertebral MOF fx (<2 yrs)—*n* (%)	4 (21)	3 (27)	6 (27)	ns
Prevalent non-MOF fractures	6 (32)	5 (45)	12 (54)	ns
Recent non-MOF fx (<2 yrs)—*n* (%)	1 (5)	1 (9)	3 (14)	ns
Comorbidities—*n* (%)	11 (58)	8 (72)	15 (68)	ns
MACE—*n* (%)	0 (0)	0 (0)	0 (0)	ns
CV RF—*n* (%)	10 (52)	5 (45)	11 (50)	ns
VTE—*n* (%)	0 (0)	2 (18)	1 (5)	ns
CKD—*n* (%)	3 (16)	2 (18)	7 (32)	ns
MGUS—*n* (%)	2 (10)	0 (0)	0 (0)	ns
Associated rheumatologic diseases—*n* (%)	7 (16)	3 (28)	10 (45)	ns
Rheumatoid arthritis	5 (26)	3 (28)	8 (37)	ns
Sjogren syndrome	1 (5)	0 (0)	2 (9)	ns
Cryoglobulinemia	1 (5)	0 (0)	0 (0)	ns
DeFRA 10y % fracture risk ± SD	48 ± 15	50 ± 14	44 ± 9	ns
FRAX 10y % fracture risk ± SD	33 ± 12	36 ± 15	31 ± 10	ns
FRAXplus adjusted for recency° 10y % fracture risk ± SD	37 ± 14	47 ± 16	33 ± 13	ns
Mean femoral neck BMD ± SD—g/cm^2^	0.618 ± 0.110	0.692 ± 0.060	0.642 ± 0.067	ns
Mean total hip BMD ± SD—g/cm^2^	0.639 ± 0.134	0.711 ± 0.058	0.740 ± 0.077	^b^
Mean lumbar spine BMD ± SD—g/cm^2^	0.842 ± 0.125	0.833 ± 0.124	0.743 ± 0.064	^a^ ^,^ ^b^
Years of denosumab treatment (IQR)	NA	4 (3–5)	3 (2–4)	ns

Abbreviations: BMD, bone mineral density; CKD, chronic kidney disease; CV RF, cardiovascular risk factor; IQR, interquartile range; MACE, major adverse cardiovascular event; MGUS, monoclonal gammopathy of undetermined significance; MOF, major osteoporotic fracture (hip, wrist, humerus); SD, standard deviation; VTE, venous thromboembolism.

a *P*-value < 0.05 denosumab alone vs romosozumab + denosumab; 1–6 months from fracture was estimated since vertebral fractures were radiographic and not clinical.

b *P*-value < 0.05 denosumab alone vs romosozumab alone.

### Romosozumab alone

#### Bone mineral density

**Figure 1:**
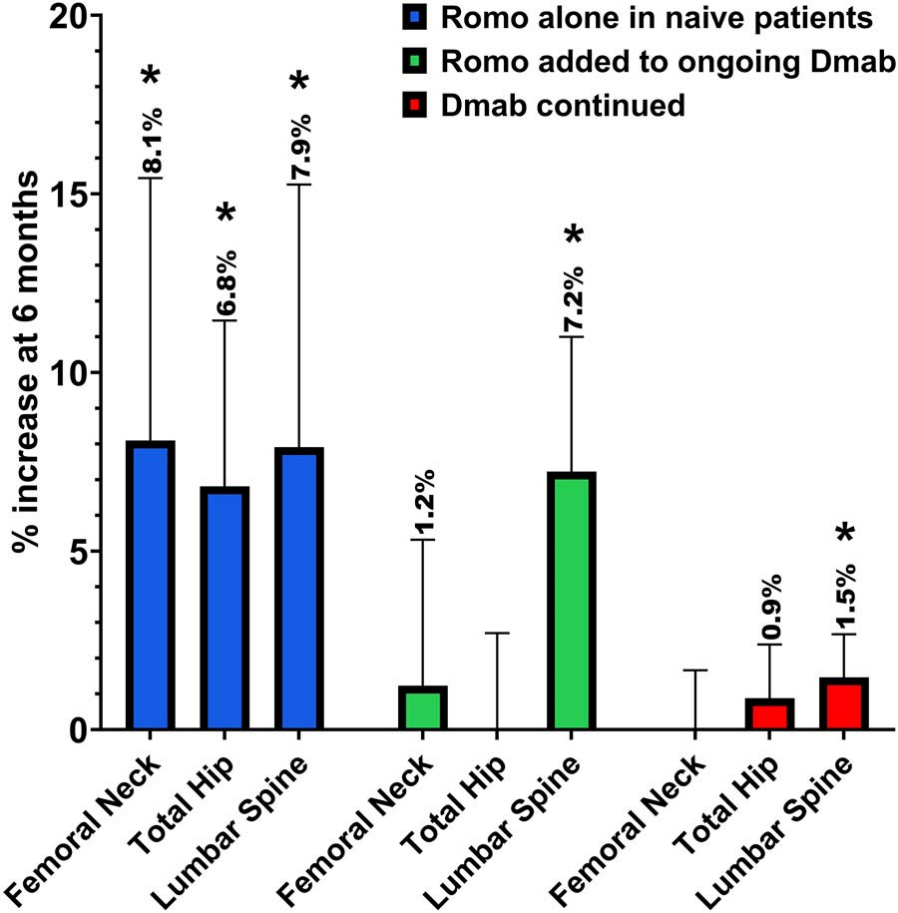
BMD percent change after 6 months of romosozumab treatment alone in naïve patients, romosozumab added to ongoing denosumab or continued denosumab. Romo, romosozumab; Dmab, denosumab; ^*^P-value < 0.01 vs baseline; for between-groups comparison, see the text.

BMD increased at all sites at 6 months of follow-up (femoral neck +8.1% SD ±10.3, total hip +6.8% SD ±6.5 and lumbar spine +7.9% SD ±11.6; p 0.034, p 0.009, and p 0.037, respectively). Percent change in BMD levels are shown in Figure 1. Absolute changes in BMD are reported in supplementary materials (Table S1 and Figure S1). No new clinical fractures were reported. No new radiographic vertebral fractures were reported.

#### Bone turnover markers and bone modulators

**Figure 2:**
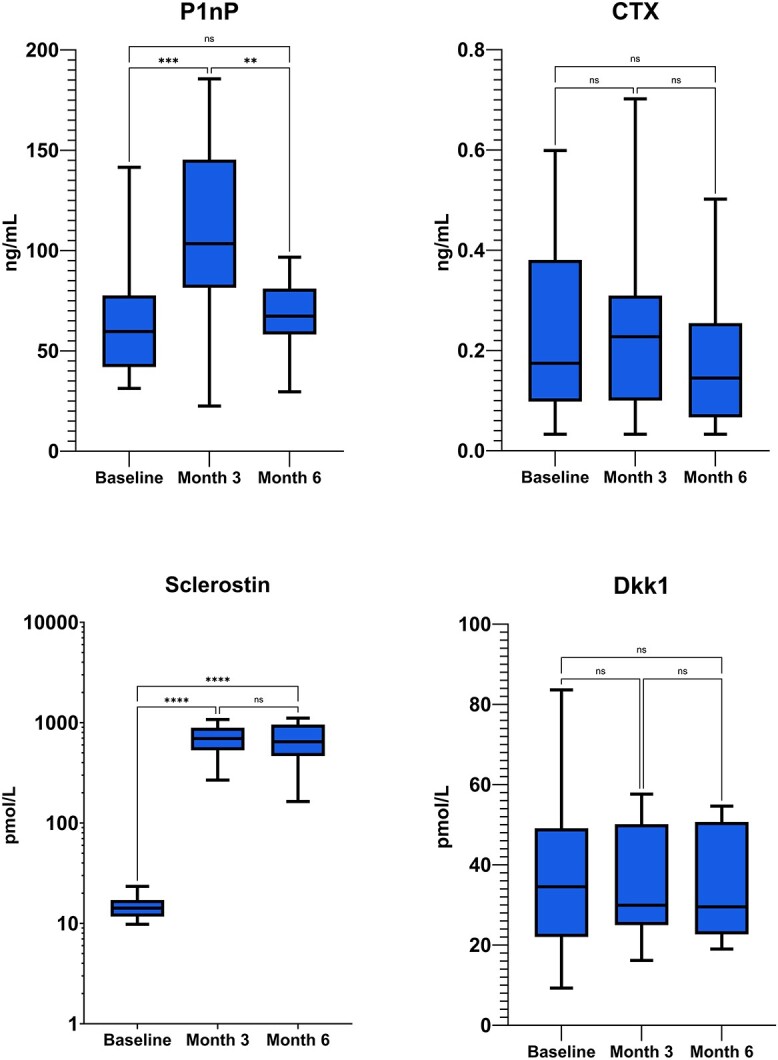
Absolute changes in bone turnover markers (P1nP and CTX) and bone modulators (Sclerostin and Dkk1) in patients treated with romosozumab alone in naïve patients. P1nP, Procollagen I Intact N-Terminal Peptide; CTX, C-terminal telopeptide of type I collagen.

We found a statistically significant increase in P1nP levels at month 3 (+70.4% SD ±64.5 p 0.0002) but not at month 6 (+19.8% SD ±54.1 p ns). CTX did not change significantly (+25.3% SD ±137.1 p ns at month 3 and –15.6% SD ±38.7 p ns). Sclerostin increased sharply at month 3 (+4927% SD ±1999 *P* < .0001) and month 6 (+4171% SD ±1965 *P* < .0001). Dkk1 levels did not change (+9.3% SD ±54.0 p ns at month 3 and + 12.5% SD ±63.1 p ns). Absolute changes in bone turnover markers and bone modulators are shown in Figure 2. Detailed percent and absolute changes are reported in supplementary materials (Table S1 and Figure S2).

#### Calcium phosphate metabolism

**Figure 3:**
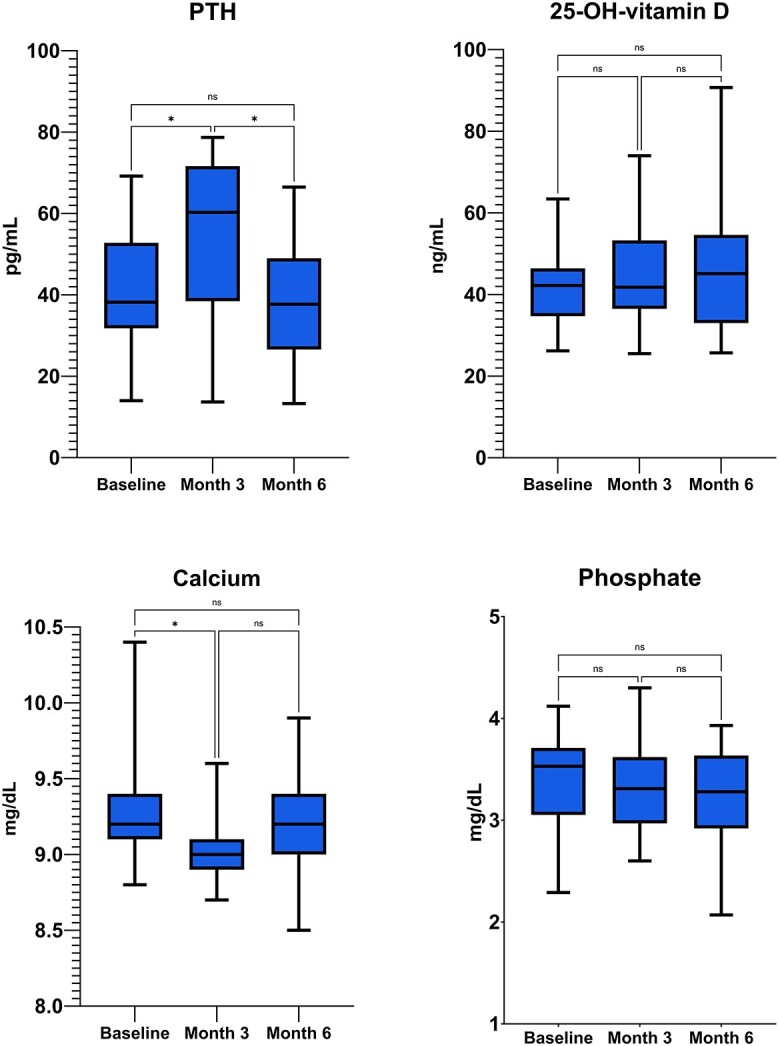
Absolute changes in calcium phosphate metabolism (calcium corrected, phosphate, 25-OH-vitamin D and parathyroid hormone) in patients treated with romosozumab alone in naïve patients. PTH, parathyroid hormone.

We found a small decline in calcium concentration at month 3 (−2.8% SD ±3.8 p 0.037), which settled back to normal concentrations at month 6 (−0.06% SD ±4.1 p ns. Concomitantly, PTH levels were found to increase at month 3 (+47.6% SD ±62.1 p 0.013) and returned back to normal at month 6 (+9.4% SD ±26.1 p ns. No statistically significant variation in phosphate was detected (−3.8% SD ±12.6 p ns at month 3 and −5.9% SD ±11.2 p ns at month 6). Vitamin D levels were sufficient during the whole duration of the study (mean 41.9 ng/mL SD ±10.1 at baseline and stable thereafter). Absolute changes in calcium phosphate metabolism are shown in Figure 3. Detailed percent and absolute changes are reported in supplementary materials (Table S1 and Figure S3).

### Combination treatment

#### Bone mineral density

We found statistically significant increase at the lumbar spine (lumbar spine +7.2% SD ±4.5; p 0.027). In contrast, we did not find any difference in BMD at femoral neck and total hip BMD (femoral neck +1.2% SD ±4.9, total hip −0.06% SD ±3.3 and, p ns). Percent change in BMD levels are shown in Figure 1. Absolute changes in BMD are reported in supplementary materials (Table S2 and Figure S4). No new clinical fractures were reported. No new radiographic vertebral fractures were reported.

#### Bone turnover markers and bone modulators

**Figure 4:**
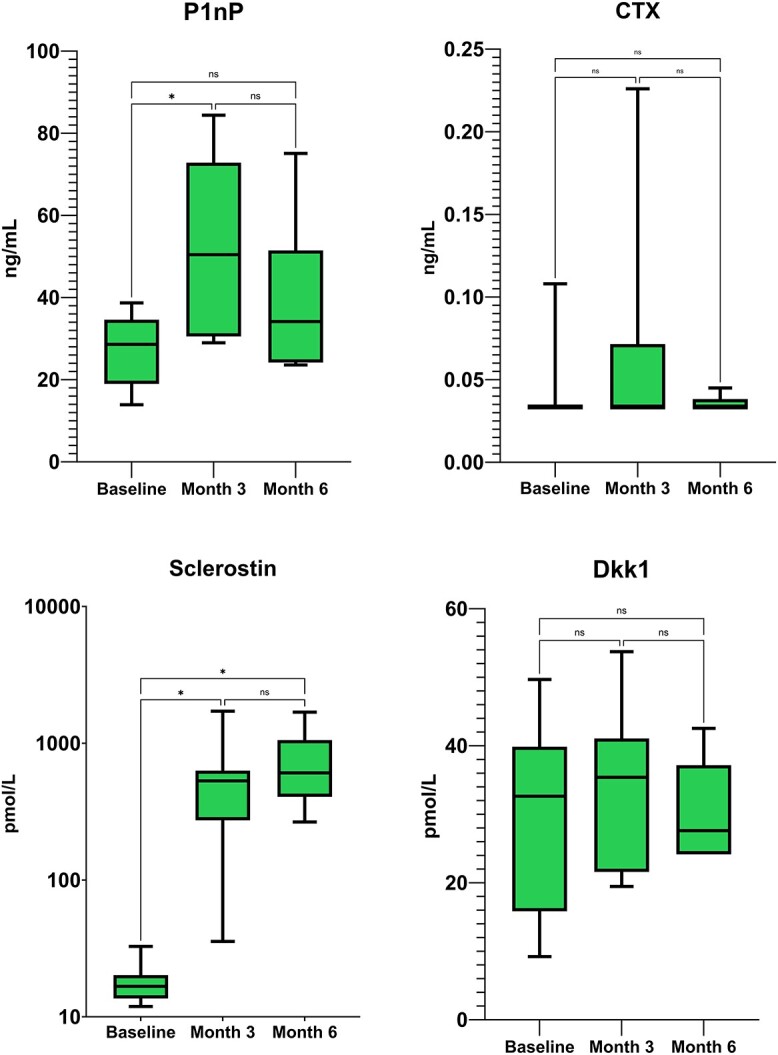
Absolute changes in bone turnover markers (P1nP and CTX) and bone modulators (Sclerostin and Dkk1) in patients treated with romosozumab added to ongoing denosumab. P1nP, Procollagen I Intact N-Terminal Peptide; CTX, C-terminal telopeptide of type I collagen.

P1NP levels were found to have increased significantly at month 3 (+99.1% SD ±100.5 p 0.027), while only a trend toward was found at month 6 (+66.9% SD ±115.1 p ns). CTX levels were markedly suppressed at all evaluations, without significant change at month 3 (+67.2% SD ±182.8 p ns) and month 6 (+4.6% SD ±7.7 p ns). Sclerostin levels showed the same overshoot as in romosozumab alone patients, with a marked increase at month 3 (+2928% SD ±1527 p 0.0009) and month 6 (+4104% SD ±1047 p 0.0001). Dkk1 levels were not found to vary significantly (+60.1% SD ±118.5 p ns at month 3 and + 74.3% SD ±166.8 p ns at month 6). Absolute change in bone turnover markers and bone modulators are shown in Figure 4. Detailed percent and absolute changes are reported in supplementary materials (Table S2 and Figure S5).

#### Calcium phosphate metabolism

**Figure 5:**
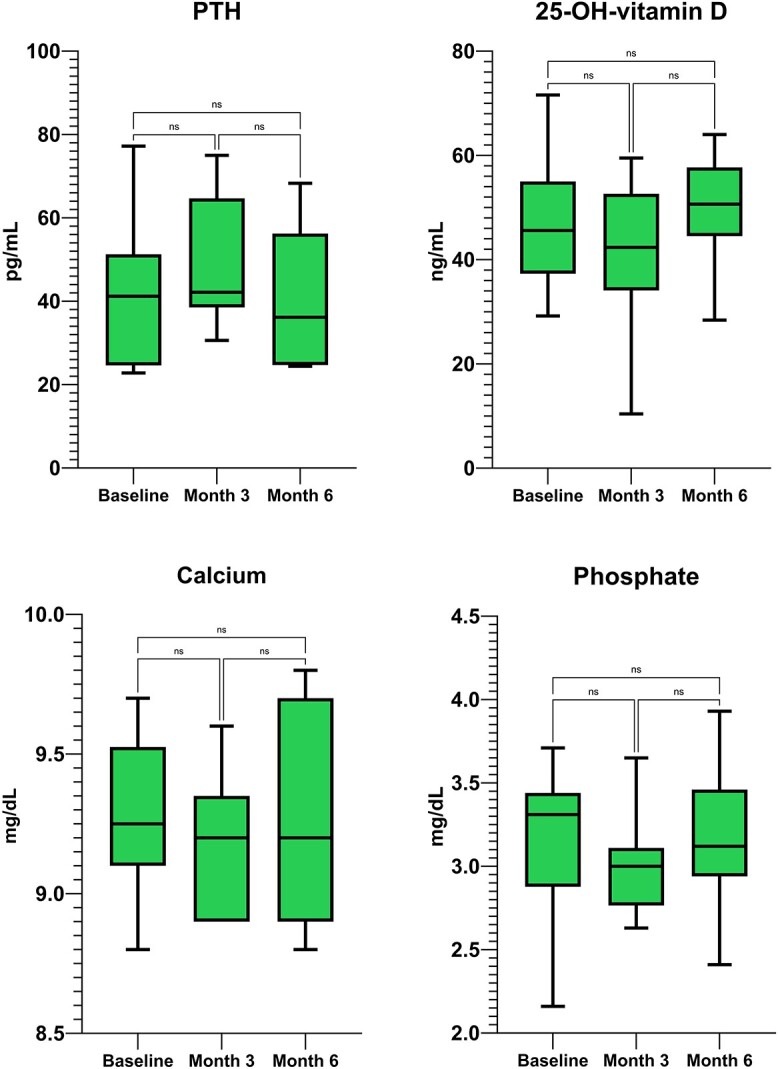
Absolute changes in calcium phosphate metabolism (calcium-corrected, phosphate, 25-OH-vitamina D and parathyroid hormone) in patients treated with romosozumab added to ongoing denosumab. PTH, parathyroid hormone.

Calcium concentration did not change significantly (−1.3% SD ±3.9 p ns at month 3 and + 1.6% SD ±5.4 p ns at month 6). PTH levels did not change significantly (+11.1% SD ±31.0 p ns at month 3 and −19.9% SD ±39.7 p ns at month 6). No statistically significant variation in phosphate was detected (−3.8% SD ±12.6 p ns at month 3 and −5.9% SD ±11.2 p ns at month 6). Vitamin D levels remained stable (mean 41.9 ng/mL SD ±10.1 at baseline and stable thereafter). Absolute changes in calcium phosphate metabolism are shown in Figure 5. Detailed percent and absolute changes are reported in supplementary materials (Table S2 and Figure S6).

### Denosumab alone

#### Bone mineral density

We found statistically significant increase at the lumbar spine (lumbar spine +1.5% SD ±2.7; p 0.022). In contrast, we did not find any difference in BMD at femoral neck and total hip BMD (femoral neck −0.4% SD ±4.8, total hip +0.9% SD ±3.4, p ns). Percent change in BMD levels are shown in Figure 1. Absolute changes in BMD are reported in supplementary materials (Table S3 and Figure S7). No new clinical fractures were reported. No new radiographic vertebral fractures were reported.

#### Bone turnover markers and bone modulators

Bone markers and modulators did not change except for a significant, small decrease in P1nP and sclerostin serum levels between baseline and month 3. Detailed percent and absolute changes are reported in supplementary materials (Table S3, Figure S8, and Figure S9).

#### Calcium phosphate metabolism

Calcium phosphate metabolism did not change at any time point. Detailed percent and absolute changes are reported in supplementary materials (Table S3, Figure S10, and Figure S11).

### Group comparisons

The changes in BMD between groups (shown in Figure 1), from baseline, were examined using a repeated measures model. Lumbar spine BMD increased significantly more in patients treated with romosozumab alone vs denosumab alone (p 0.007) and in patients treated with romosozumab combined with denosumab vs denosumab alone (p 0.041). We find a significant difference in femoral neck BMD between romosozumab alone vs denosumab alone (p 0.002) but not between romosozumab alone and romosozumab combined with denosumab. Total hip BMD increased more in romosozumab alone vs the two other groups (p 0.023 against romosozumab combined to denosumab and p 0.015 against denosumab alone).

### Correlations analysis

After accounting for multiplicity using the FDR approach with the two-stage step-up method of Benjamini, Krieger, and Yekutieli (*Q* value 5% of FDR), we found a significant positive association between delta P1nP between baseline and M6 and delta femoral neck BMD between baseline and month 6 in patients receiving romosozumab alone (*r*^2^ 0.652, p 0.008, Figure S7) and between baseline sclerostin levels and delta femoral neck BMD between baseline and month 6 in patients receiving romosozumab alone (*r*^2^ 0.408, p 0.047, Figure S8).

## Discussion

We conducted an observational prospective study on the effectiveness of romosozumab either alone or in combination with denosumab in postmenopausal women at high risk of fracture. To our knowledge, this is the first report of combined romosozumab and denosumab.

In summary, we found that romosozumab added to ongoing treatment with denosumab was associated with a significant increase in P1nP serum levels, which, in turn, led to a significant increase in lumbar spine BMD, greater than the increase seen with continued denosumab. However, we found that femoral neck and total hip BMD did not increase in both the combination and denosumab alone groups. The observed blunted effect at the femur is challenging to explain. One hypothesis is that patients receiving long-term denosumab may have already developed a higher cortical BMD, potentially limiting the extent of increase attainable with subsequent romosozumab treatment. On the other hand, endocortical modeling–based bone formation might have been somehow attenuated by long-term denosumab treatment.^5^

P1nP increased markedly both in the romosozumab alone group and in the combination group. Moreover, the difference in P1NP levels between baseline and month 6 was found to be positively associated with femoral neck BMD delta, indicating a robust effect of romosozumab on modeling bone formation.^21^ For the first time, we demonstrated that romosozumab was able to stimulate bone formation even in patients with a closed resorption phase, as evidenced by long-term denosumab treatment and markedly suppressed bone turnover. Remarkably, previous studies showed that treatment with denosumab or bisphosphonates was associated with a considerable and dose-dependent increase in serum sclerostin.^22–24^

We found a steep increase in sclerostin levels in all our patients receiving romosozumab, already after 3 months of therapy. The sharp rise of sclerostin is too uniform to be considered an actual measure of the serum sclerostin levels of the patients. We interpret these data as interference from romosozumab itself with the fully automated CLIA test we used. Bioactive sclerostin could be a more accurate marker to measure to correctly evaluate sclerostin levels: Further studies are needed to corroborate this hypothesis. The dosage of other markers (such as RANKL and OPG) could also provide clues on how to interpret the high sclerostin levels. Further analysis and a 12-month study are ongoing.

We demonstrated that basal sclerostin levels were positively associated with femoral neck BMD increase. Therefore, measuring basal sclerostin levels could prove useful to predict the response to therapy.

We did not find any change in Dkk1 serum levels during the follow-up of our patients. This result cannot be fully explained and was somehow unexpected. We would have anticipated observing an increase in Dkk1 to compensate for the suppression of sclerostin. Indeed, the expression of Dkk1 in bone tissue was observed to rise in both SOST knockout mice and rats treated with anti-sclerostin antibodies,^25^ mirroring the Dkk1 increase in individuals affected by sclerostosis.^26^ Our results did not confirm this hypothesis, and probably other mechanisms that limit and plateau bone formation are ongoing during romosozumab treatment.

Calcium levels were found to transiently decrease at month 3 in the romosozumab alone group. This finding was somewhat expected, as romosozumab not only has potent anabolic activity but also possesses antiresorptive action. Indeed, the transient calcium decline (mild and asymptomatic) can be easily explained by the marked stimulation of both osteoblasts with concomitant relative inhibition of osteoclasts. This phenomenon, previously described in the phase 1 study of romosozumab,^27^ also explains the transient increase in PTH we found. In contrast, calcium and PTH levels did not change significantly during the combination of romosozumab and denosumab. Indeed, the homeostasis of calcium during denosumab treatment is almost entirely dependent on calcium handling in the kidneys, and the osteoclasts, whose activity is markedly suppressed, cannot further contribute to decreasing serum calcium levels. Our results could provide a hint of the safety of the combination treatment.

Other combination therapies had been studied in the past. The combination of teriparatide and denosumab was also demonstrated to be efficacious in the DATA study.^11^ However, the DATA study enrolled naïve patients, and to date, no other study has tested the efficacy of an anabolic/dual action treatment added to ongoing denosumab.^11^ The closest approximation to such a study (anabolic added to ongoing and continued denosumab) was conducted in 2016 by our group.^28^ In this study, patients were treated with denosumab first, and teriparatide was added 3 months later. P1nP levels increased 3 months after the beginning of the anabolic agent, paralleling the results of the present study. However, a 3-month on denosumab period might not be enough to close all remodeling units.^2^^,^^4^ In addition, the reversal phase might not have been ended yet.^29^ The core concept is indeed that, in both the DATA study and the 2016 study by our group, patients were essentially naive to any anti-osteoporotic treatment.

The add-on therapy of teriparatide with ongoing long-term (>2 years) denosumab has not been studied yet. Nonetheless, we can speculate that such treatment might be suboptimal due to the deep suppression of bone turnover that could blunt most of the effect on remodeling bone formation and overflow remodeling bone formation. In contrast, we showed that the effect of romosozumab on trabecular bone might not be affected by ongoing denosumab.

Interestingly, we can also speculate that the combination of romosozumab and denosumab in naïve patients might not translate into additional benefits compared to romosozumab alone. As a matter of fact, romosozumab has a notable anti-resorptive effect itself. It is likely that adding a potent anti-resorptive would not provide any additional benefit (ie, it would not increase the bone anabolic window). In contrast, the combination therapy of teriparatide and denosumab in naïve patients has been proven to increase BMD faster and to a greater extent compared to either drug alone. In this scenario, the anabolic window is greatly improved.

Our study should be interpreted in the light of its strengths and limitations. The study is certainly limited by the sample size and the observational nature without formal randomization. This absence of randomization has led to inherent differences in fracture profiles between the study groups. Despite this, there were no significant differences in all other relevant parameters measured. However, it is important to recognize that the distinct fracture profile, particularly the higher incidence of recent vertebral fractures in the combination group, may impact the comparability of these groups. In addition, we had only the 6-month data available, but we are planning to extend the study also to the 12-month period. Indeed, denosumab has been proven to increase BMD (even at the femur) at each yearly timepoint for up to 10 years in the FREEDOM extension study. We did not find a significant increase at the femur at 6 months, but again, this finding might be related to the short follow-up and to the scarcity of the sample size. Our study was not indeed powered for detecting small differences in BMD or for fracture outcome.

In conclusion, the study we conducted in our cohort demonstrated that romosozumab led to an increase in P1NP serum levels and lumbar spine BMD in patients already on denosumab. However, the positive effect on bone formation was somehow blunted at the femur. Nonetheless, our data might support the addition of romosozumab to ongoing denosumab treatment. A randomized controlled trial with longer follow-up and powered to explore fracture data is needed to confirm our results.

## Supplementary Material

supplementary_materials_ziae016

## Data Availability

Data of the analysis are available upon reasonable request.
