# Supplementary material for: Romosozumab added to ongoing denosumab in postmenopausal osteoporosis, a prospective observational study
Source: JBMR Plus. 2024 Feb 7;8(4):ziae016. doi: 10.1093/jbmrpl/ziae016 (PMC10972672; doi:10.1093/jbmrpl/ziae016)

**Supplementary materials**

**Romosozumab and denosumab combination therapy in postmenopausal osteoporosis**

**Authors:** Giovanni Adami^1^, Elisa Pedrollo^1^, Maurizio Rossini^1^, Angelo Fassio^1^, Vania Braga^1^, Camilla Benini^1^, Ombretta Viapiana^1^, Davide Gatti**^1^**

1. Rheumatology Unit, Azienda Ospedaliera Universitaria Integrata di Verona, Verona, Italy

**Table of content**

**Table s1.** Calcium phosphate metabolism, bone turnover markers and bone modulators markers during romosozumab treatment alone

**Table s2.** Calcium phosphate metabolism, bone turnover markers and bone modulators markers during romosozumab added to ongoing denosumab

**Table s3.** Calcium phosphate metabolism, bone turnover markers and bone modulators markers during ongoing denosumab treatment

**Figure s1.** Absolute change in bone mineral density during romosozumab treatment alone

**Figure s2.** Percent change in bone turnover markers and modulators during romosozumab treatment alone

**Figure s3.** Percent change in calcium phosphate metabolism during romosozumab treatment alone

**Figure s4.** Absolute change in bone mineral density during romosozumab added to ongoing denosumab

**Figure s5.** Percent change in bone turnover markers during romosozumab added to ongoing denosumab

**Figure s6.** Percent change in calcium phosphate metabolism during romosozumab added to ongoing denosumab

**Figure s7.** Absolute change in bone mineral density during ongoing denosumab

**Figure s8.** Absolute change in bone turnover markers during ongoing denosumab

**Figure s9.** Percent change in bone turnover markers during ongoing denosumab

**Figure s10.** Absolute change in calcium phosphate metabolism ongoing denosumab

**Figure s11.** Percent change in calcium phosphate metabolism ongoing denosumab

**Figure s12.** Correlation between delta P1nP between baseline and M6 and delta femoral neck BMD between baseline and month 6 in patients receiving romosozumab alone

**Figure s13.** Correlation between baseline sclerostin levels and delta femoral neck BMD between baseline and month 6 in patients receiving romosozumab alone

**Table s1.** Calcium phosphate metabolism, bone turnover markers and bone modulators markers during romosozumab treatment alone

|  | Month | | | p value | | |
| --- | --- | --- | --- | --- | --- | --- |
|  | M0 | M3 | M6 | M0-M3 | M0-M6 | M3-M6 |
| Mean Ca corr ±SD – mg/dL | 9.3±0.37 | 9.02±0.22 | 9.2±0.33 | 0.0374 | ns | 0.0356 |
| Mean P ±SD – mg/dL | 3.44±0.44 | 3.32±0.48 | 3.26±0.51 | ns | ns | ns |
| Mean CTX ±SD – ng/mL | 0.241±0.179 | 0.242±0.190 | 0.177±0.146 | ns | ns | ns |
| Mean DKK1 ±SD – pmol/L | 36.08±18.29 | 34.64±13.94 | 33.76±12.91 | ns | ns | ns |
| Mean Sost ±SD – pmol/L | 14.85±3.49 | 693.6±227.9 | 660.4±274.6 | <0.0001 | <0.0001 | ns |
| Mean P1nP ±SD – ng/mL | 63.29±27.61 | 107.5±40.78 | 67.38±18.81 | 0.0002 | ns | 0.006 |
| Mean PTH ±SD – pg/mL | 40.14±13.72 | 54.16±19.25 | 38.58±15.14 | 0.0146 | ns | 0.012 |
| Mean neck BMD ±SD – g/cm^3^ | 0.618±0.110 |  | 0.667±0.132 | na | 0.0248 | na |
| Mean total hip BMD ±SD – g/cm^3^ | 0.639±0.134 |  | 0.680±0.143 | na | 0.0194 | na |
| Mean lumbar BMD ±SD – g/cm^3^ | 0.842±0.125 |  | 0.907±0.148 | na | 0.0314 | na |

**Table s2.** Calcium phosphate metabolism, bone turnover markers and bone modulators markers during romosozumab added to ongoing denosumab

|  | Month | | | p value | | |
| --- | --- | --- | --- | --- | --- | --- |
|  | M0 | M3 | M6 | M0-M3 | M0-M6 | M3-M6 |
| Mean Ca corr ±SD – mg/dL | 9.3±0.67 | 9.0±0.2 | 9.2±0.3 | ns | ns | ns |
| Mean P ±SD – mg/dL | 3.15±0.46 | 2.99±0.30 | 3.19±0.48 | ns | ns | ns |
| Mean CTX ±SD – ng/mL | 0.040±0.02 | 0.064±0.063 | 0.036±0.004 | ns | ns | ns |
| Mean DKK1 ±SD – pmol/L | 30.2±13.48 | 33.52±11.57 | 30.24±7.29 | ns | ns | ns |
| Mean Sost ±SD – pmol/L | 17.92±6.16 | 567±457.3 | 743.9±500.4 | 0.0151 | 0.0349 | ns |
| Mean P1nP ±SD – ng/mL | 27.29±8.41 | 52.02±21.35 | 39.17±19.11 | 0.0101 | ns | ns |
| Mean PTH ±SD – pg/mL | 42.51±16.79 | 49.69±15.39 | 40.33±18.17 | ns | ns | ns |
| Mean neck BMD ±SD – g/cm^3^ | 0.692±0.060 |  | 0.701±0.068 | na | ns | na |
| Mean total hip BMD ±SD – g/cm^3^ | 0.711±0.058 |  | 0.711±0.071 | na | ns | na |
| Mean lumbar BMD ±SD – g/cm^3^ | 0.833±0.124 |  | 0.889±0.109 | na | 0.001 | na |

**Table s3.** Calcium phosphate metabolism, bone turnover markers and bone modulators markers during ongoing denosumab treatment

|  | Month | | | p value | | |
| --- | --- | --- | --- | --- | --- | --- |
|  | M0 | M3 | M6 | M0-M3 | M0-M6 | M3-M6 |
| Mean Ca corr ±SD – mg/dL | 9.2±0.34 | 9,2±0,25 | 9.2±0.37 | ns | ns | ns |
| Mean P ±SD – mg/dL | 3.0±0.40 | 3.1±0.29 | 3.1±0.45 | ns | ns | ns |
| Mean CTX ±SD – ng/mL | 0.105±0.05 | 0.074±0.05 | 0.081±0.04 | ns | ns | ns |
| Mean DKK1 ±SD – pmol/L | 20.2±13.13 | 23.6±13.60 | 23.7±11.36 | ns | ns | ns |
| Mean Sost ±SD – pmol/L | 38.5±13.05 | 34.26±14.47 | 37.0±15.55 | 0.045 | ns | ns |
| Mean P1nP ±SD – ng/mL | 20.8±2.26 | 20.7±2.16 | 20.1±2.18 | ns | ns | 0.026 |
| Mean PTH ±SD – pg/mL | 25.3±10.57 | 30.3±9.09 | 26.9±11.95 | ns | ns | ns |
| Mean neck BMD ±SD – g/cm^3^ | 0.642±0.067 |  | 0.638±0.067 | na | ns | na |
| Mean total hip BMD ±SD – g/cm^3^ | 0.740±0.077 |  | 0.746±0.078 | na | ns | na |
| Mean lumbar BMD ±SD – g/cm^3^ | 0.743±0.064 |  | 0.754±0.070 | na | 0.0155 | na |

**Figure s1.** Absolute change in bone mineral density during romosozumab treatment alone


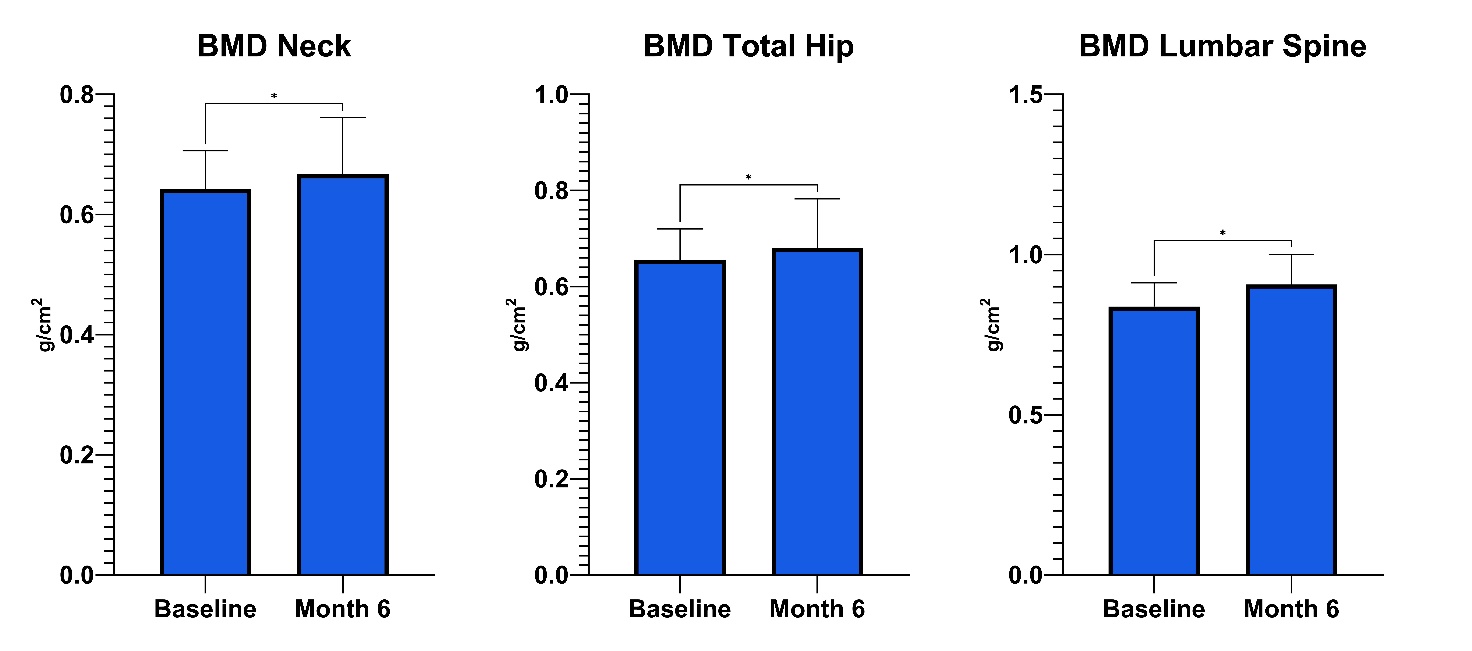


**Figure s2.** Percent change in bone turnover markers and modulators during romosozumab treatment alone


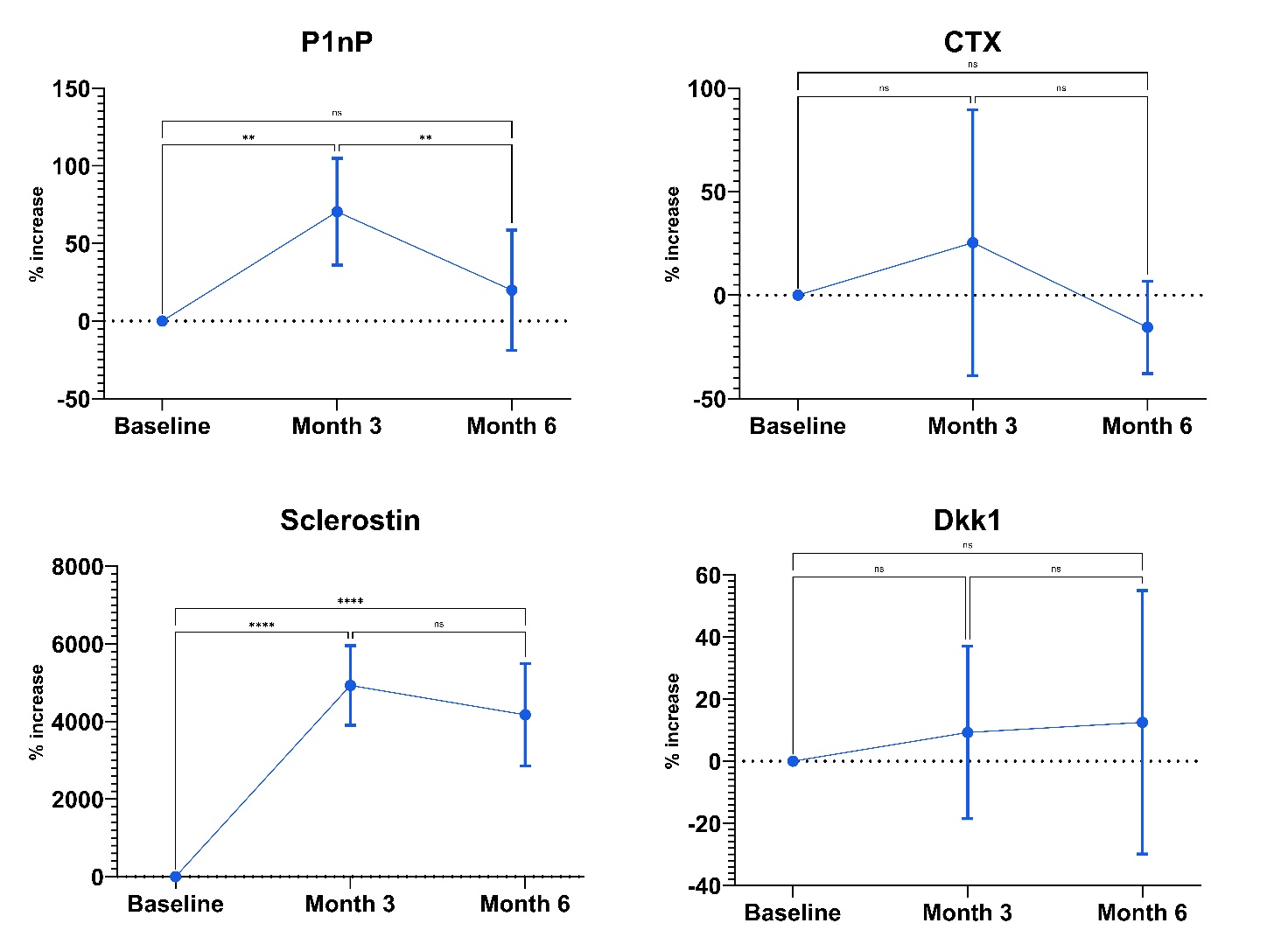


**Figure s3.** Percent change in calcium phosphate metabolism during romosozumab treatment alone


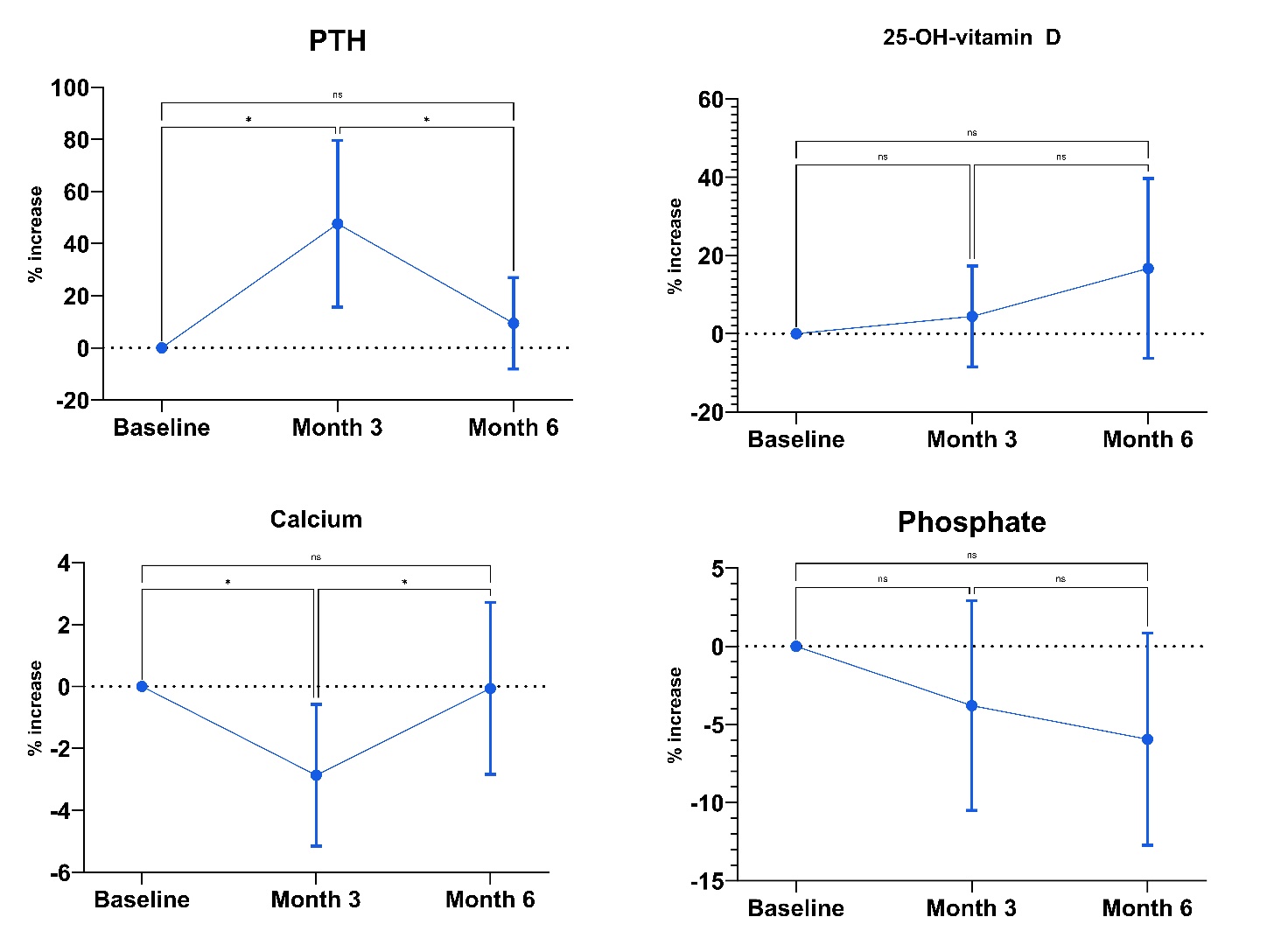


**Figure s4.** Absolute change in bone mineral density during romosozumab added to ongoing denosumab


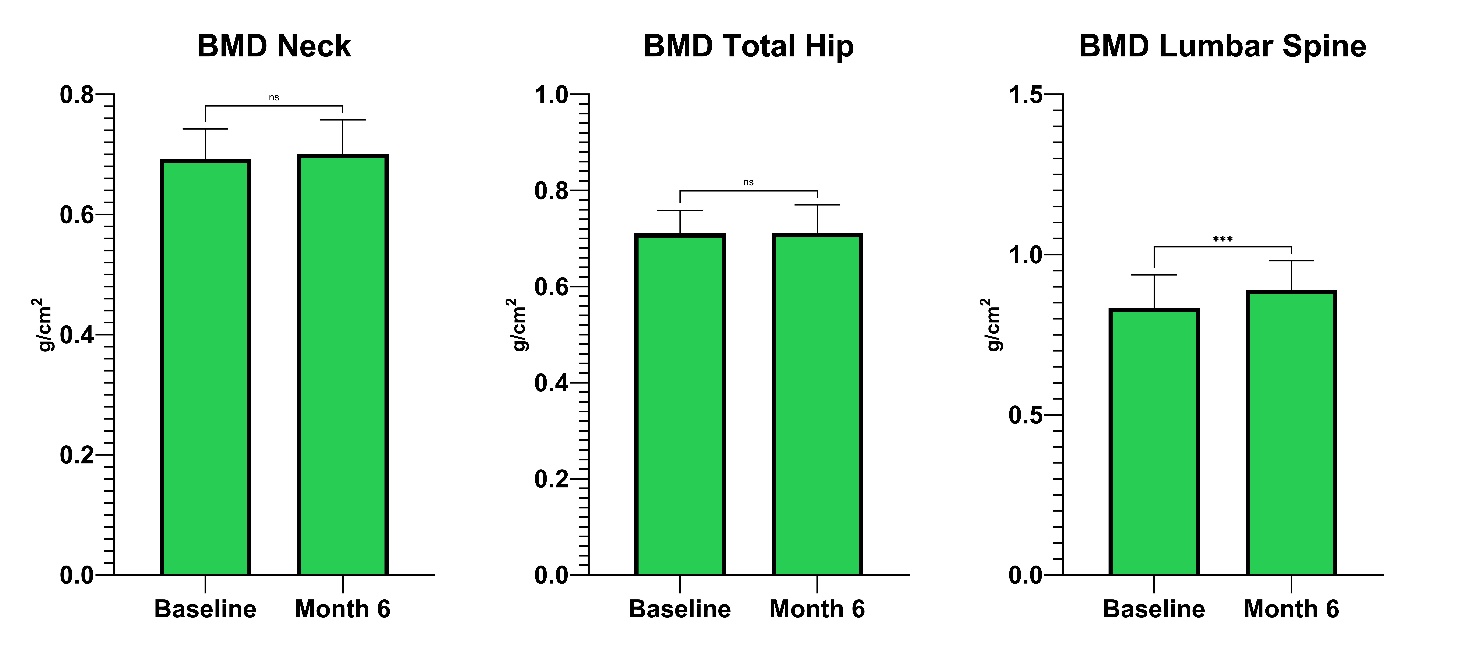


**Figure s5.** Percent change in bone turnover markers during romosozumab added to ongoing denosumab


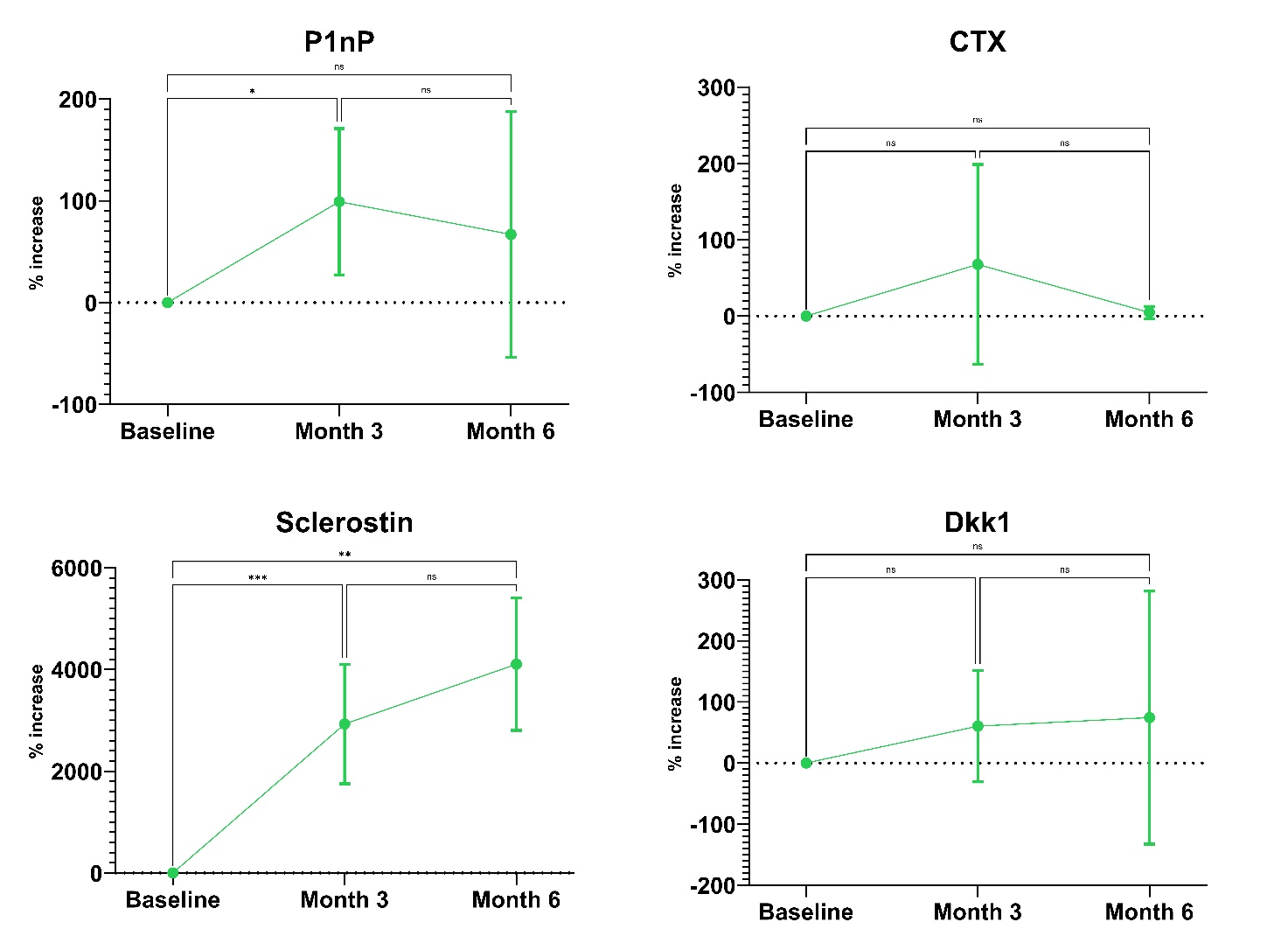


**Figure s6.** Percent change in calcium phosphate metabolism during romosozumab added to ongoing denosumab


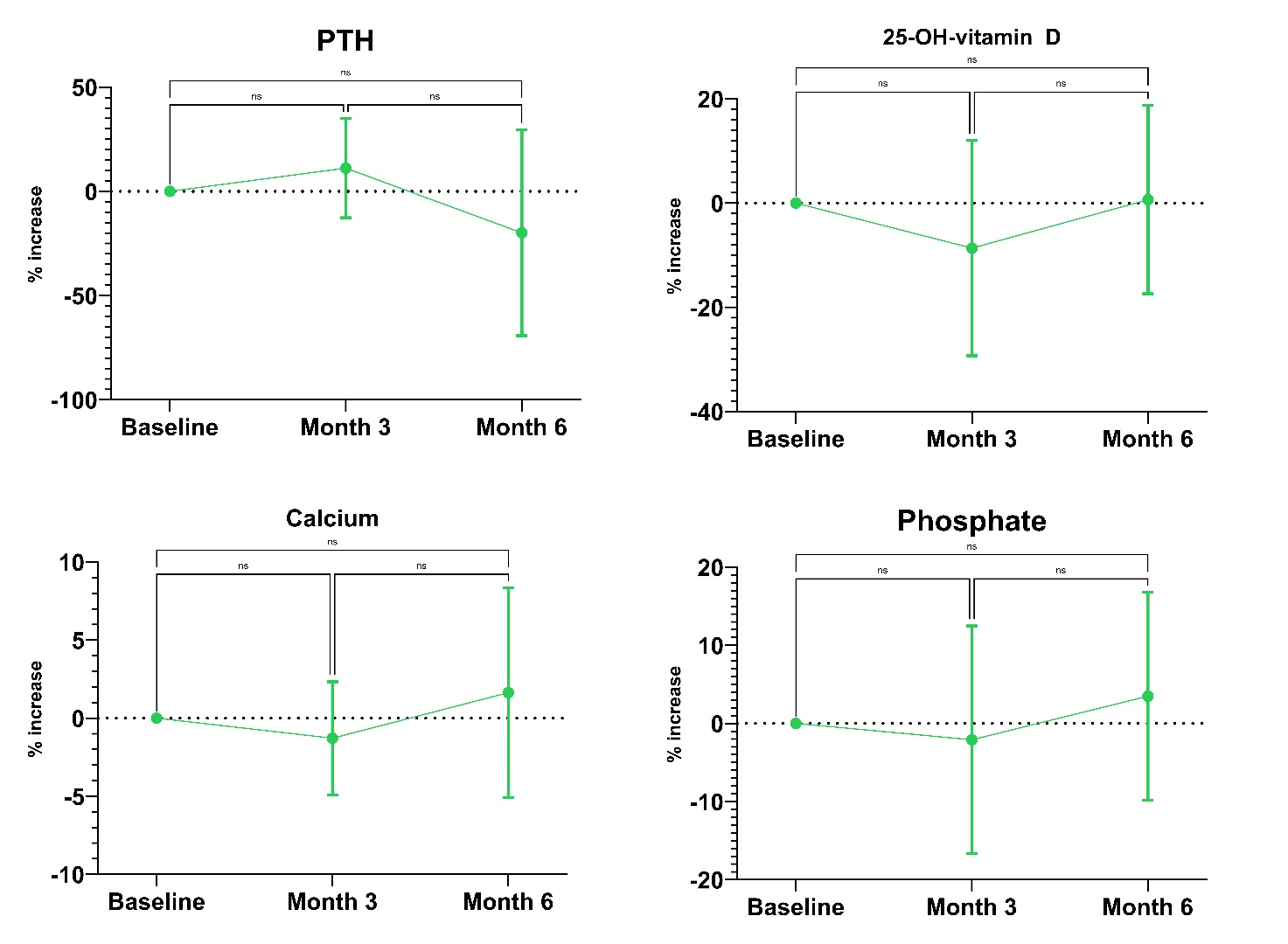


**Figure s7.** Absolute change in bone mineral density during ongoing denosumab


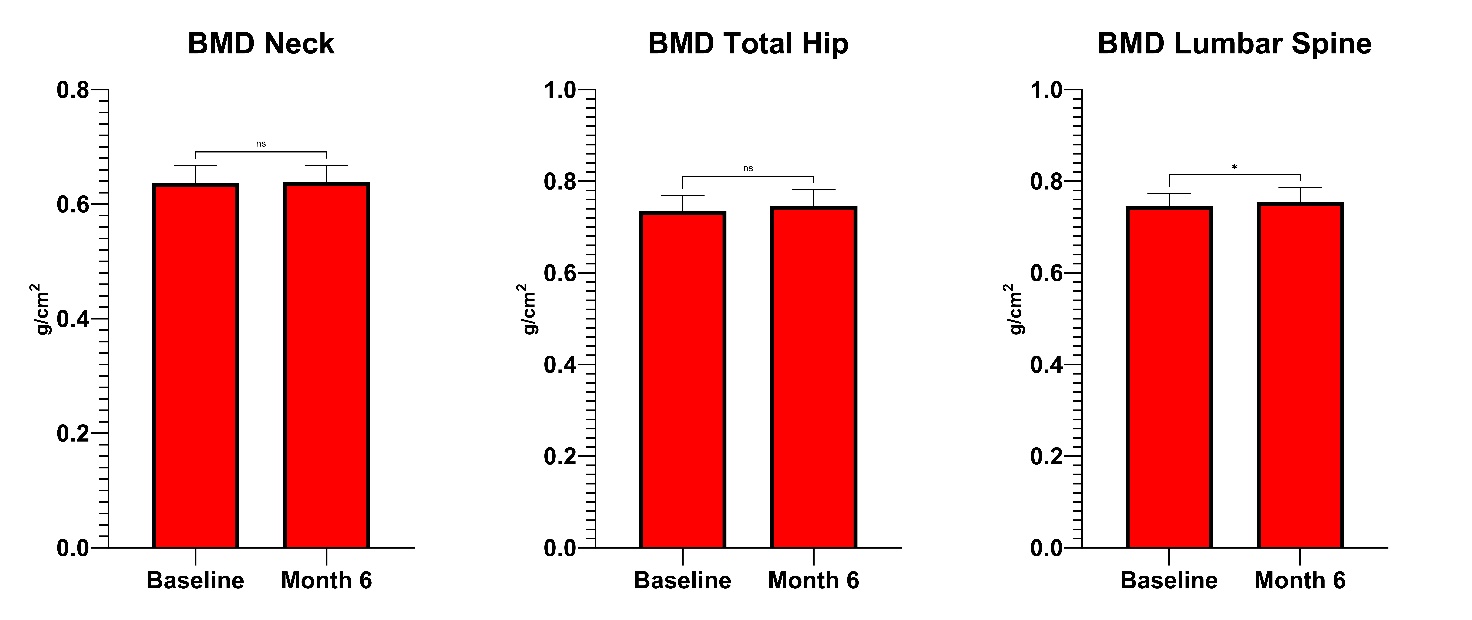


**Figure s8.** Absolute change in bone turnover markers during ongoing denosumab


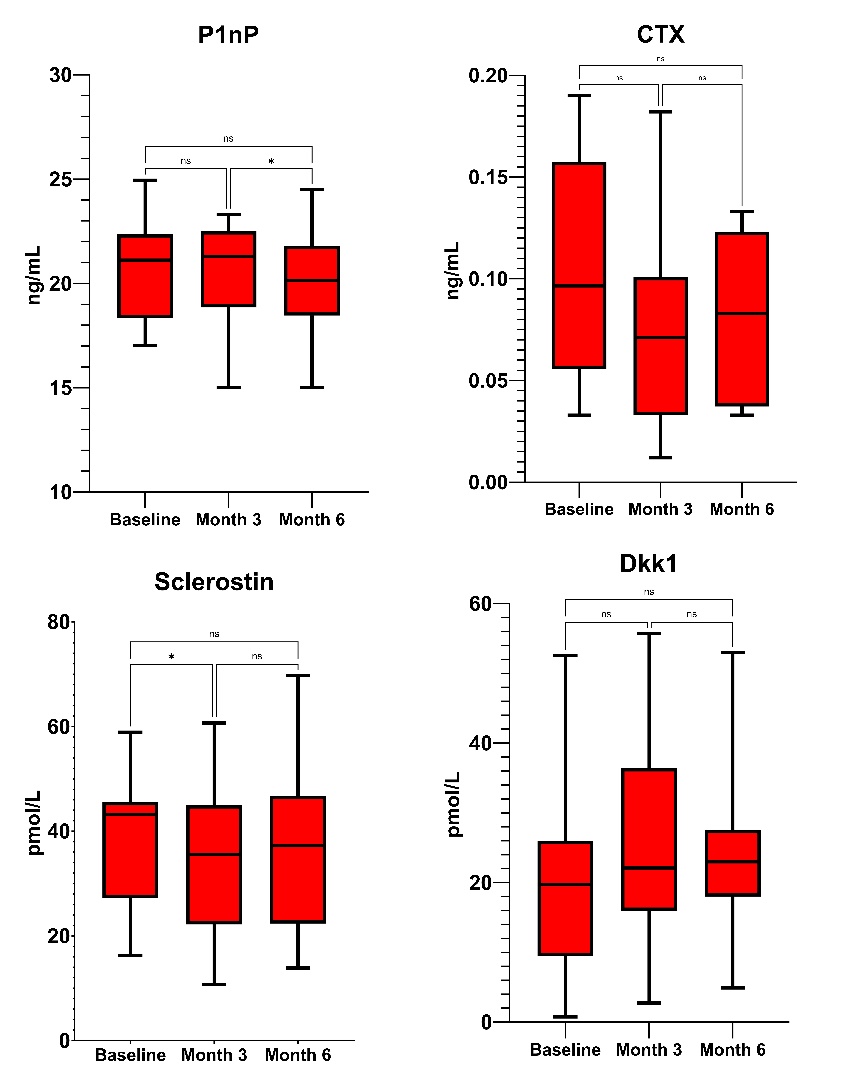


**Figure s9.** Percent change in bone turnover markers during ongoing denosumab


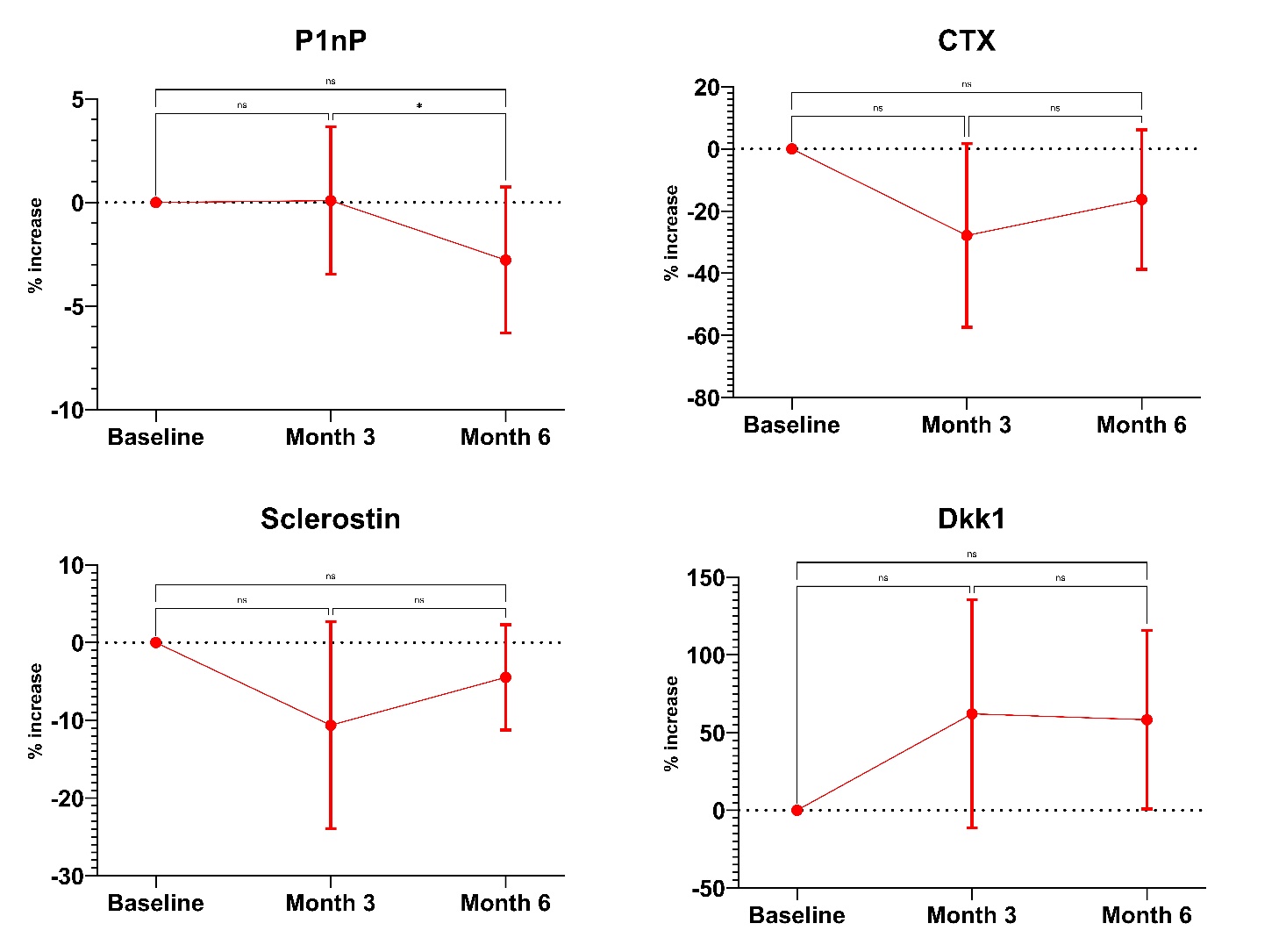


**Figure s10.** Absolute change in calcium phosphate metabolism ongoing denosumab


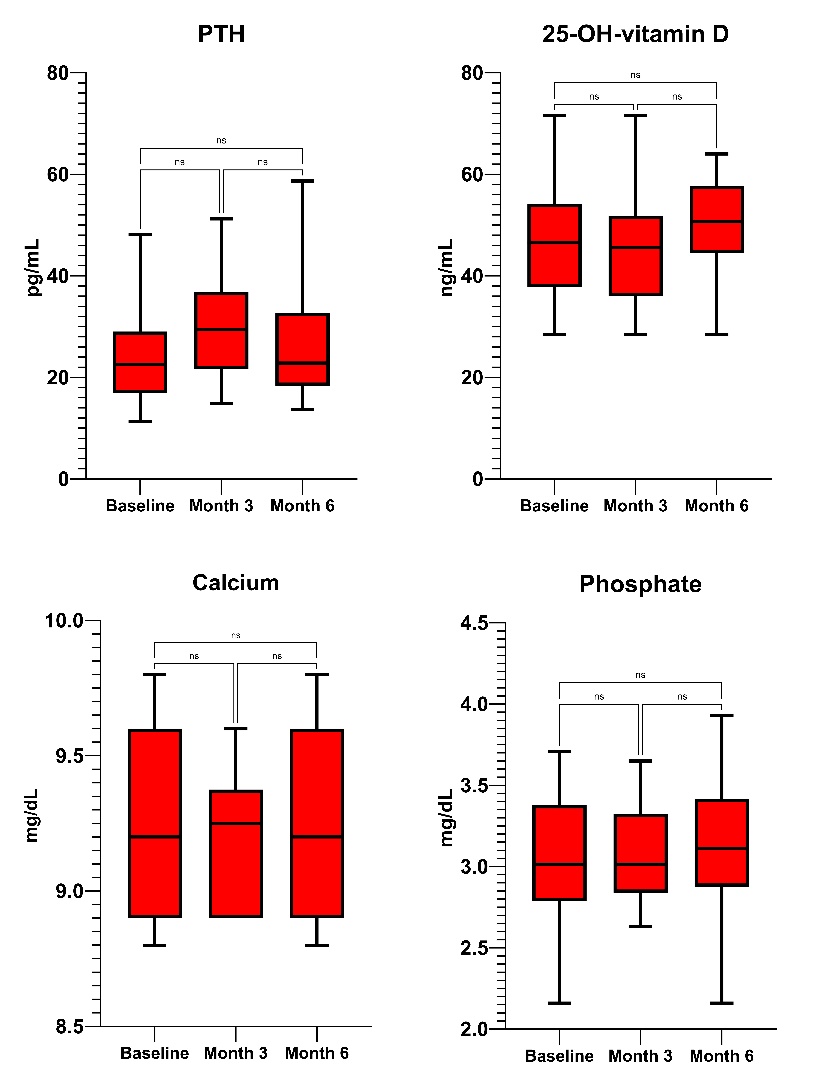


**Figure s11.** Percent change in calcium phosphate metabolism ongoing denosumab

**
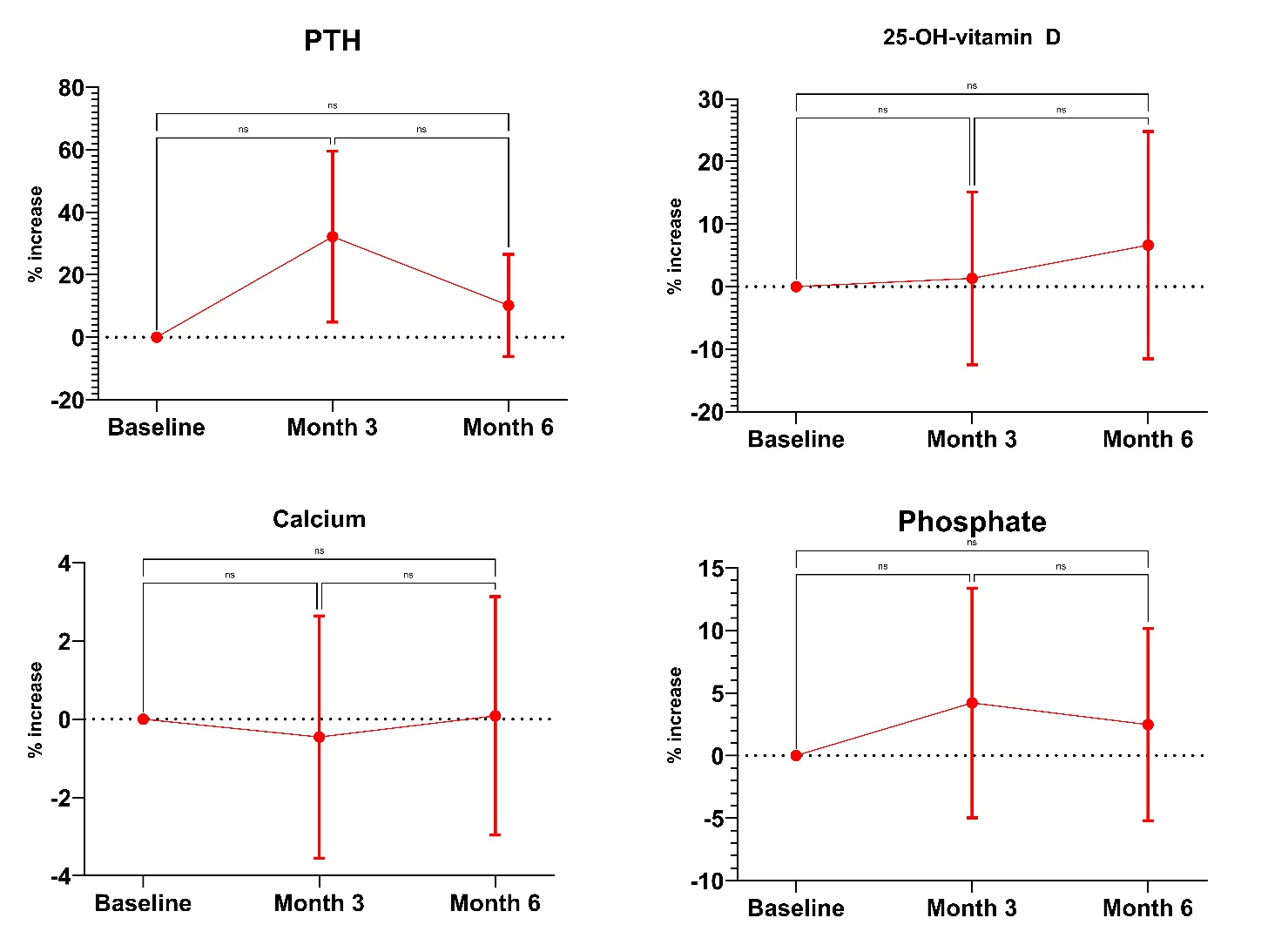
**

**Figure s12.** Correlation between delta P1nP between baseline and M6 and delta femoral neck BMD between baseline and month 6 in patients receiving romosozumab alone


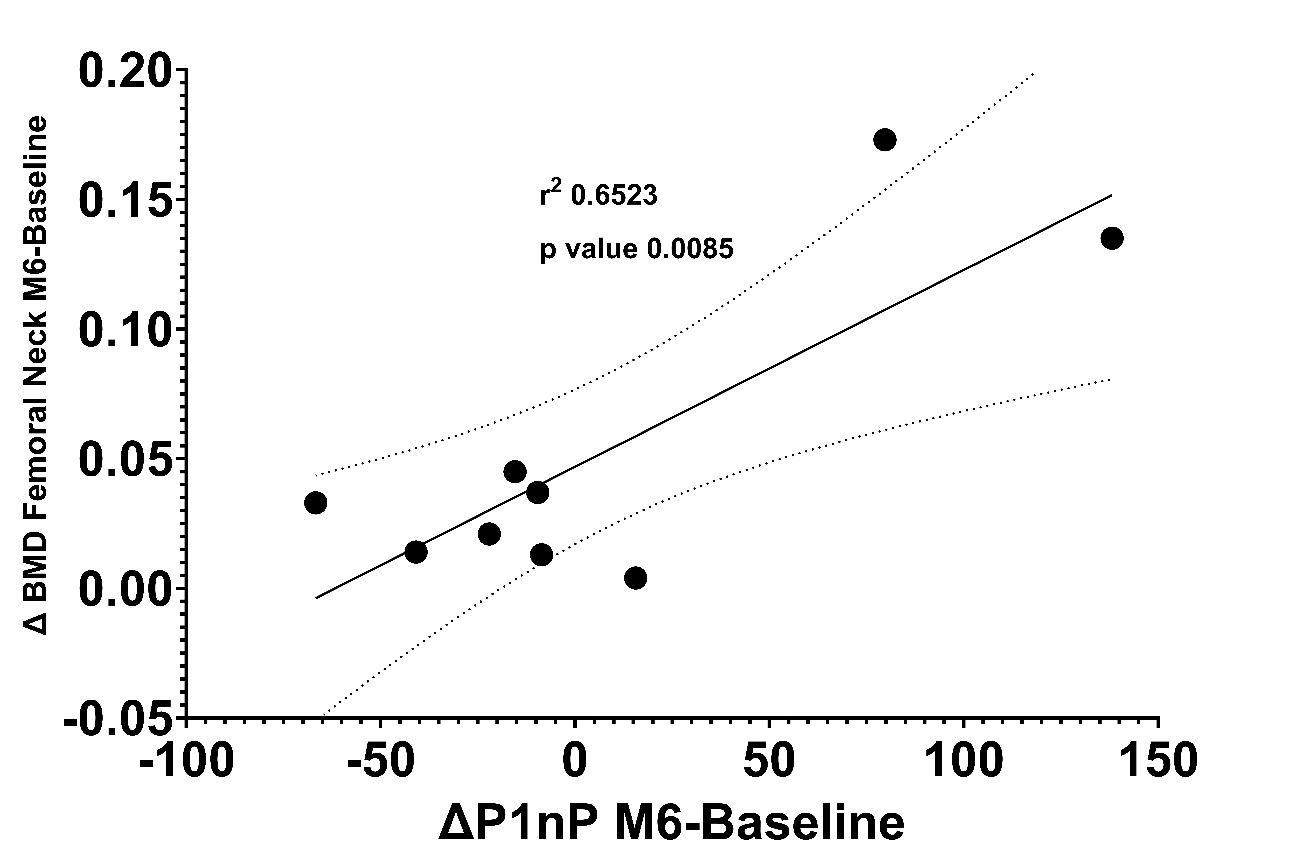


**Figure s13.** Correlation between baseline sclerostin levels and delta femoral neck BMD between baseline and month 6 in patients receiving romosozumab alone


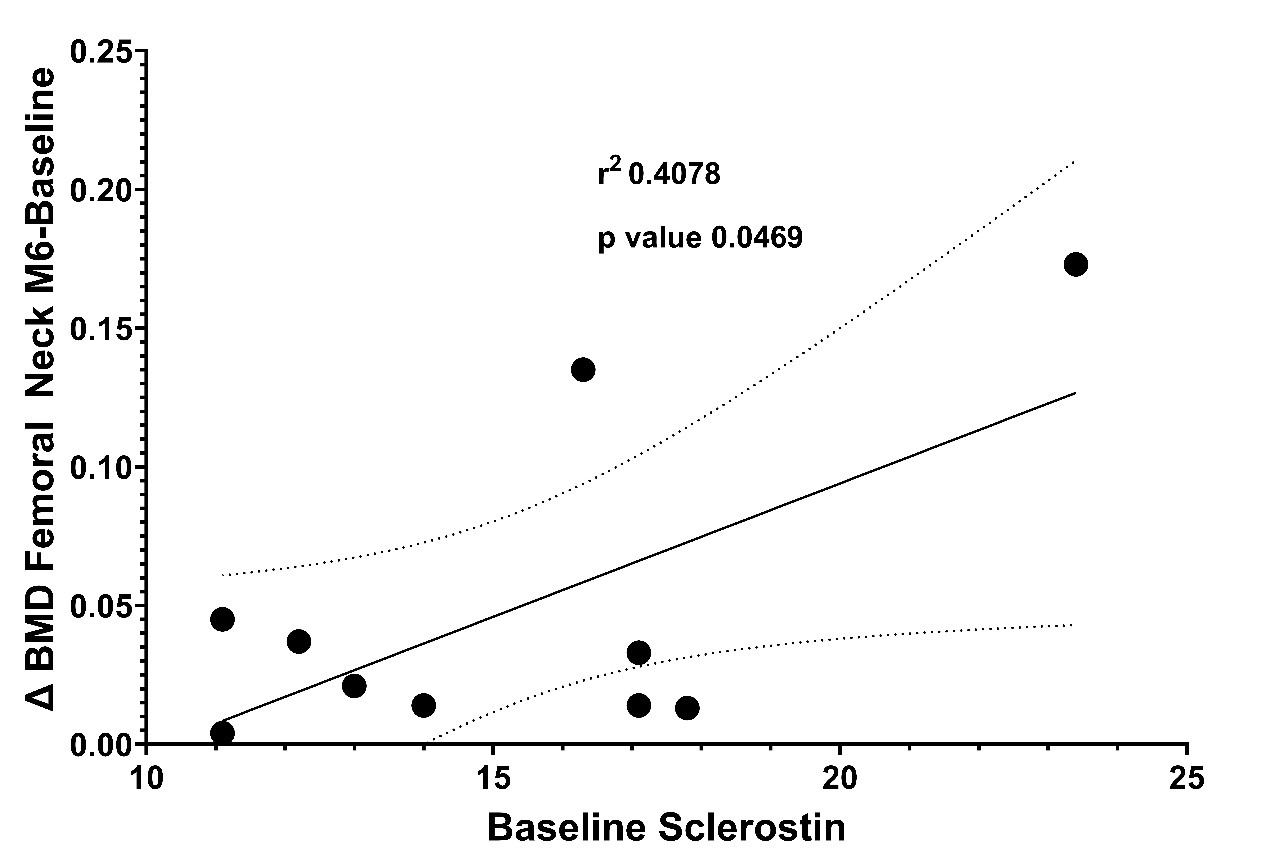

Supplement: supplementary_materials_ziae016 [file supplementary_materials_ziae016.zip]
